# The Bacterial and Fungal Microbiota of “Robiola di Roccaverano” Protected Designation of Origin Raw Milk Cheese

**DOI:** 10.3389/fmicb.2021.776862

**Published:** 2022-01-31

**Authors:** Federica Biolcati, Ilario Ferrocino, Maria Teresa Bottero, Alessandra Dalmasso

**Affiliations:** ^1^Dipartimento di Scienze Veterinarie, Università di Torino, Grugliasco, Italy; ^2^Dipartimento di Scienze Agrarie, Forestali e Alimentari, Università di Torino, Grugliasco, Italy

**Keywords:** cheese, mycobiota, metagenetic high-throughput sequencing, Robiola di Roccaverano, bacterial microbiota

## Abstract

Robiola di Roccaverano is an artisanal Protected Designation of Origin (PDO) soft cheese made with raw goat’s milk and by the addition of Natural Milk Culture (NMC) to drive the fermentation process. Cheeses collected from five different dairy plants were analyzed for their bacterial and fungal microbiota diversity. *Lactococcus lactis* and *Leuconostoc mesenteroides* were the main bacterial population, while *Galactomyces candidum* and *Kluyveromyces marxianus* constituted the core mycobiota but many other minor taxa were observed, suggesting a high level of complexity in fungal composition by these cheeses compared to bacteria population.

## Introduction

Robiola di Roccaverano is a Protected Designation of Origin (PDO) soft cheese produced with raw goat’s milk, in the Roccaverano area of the Piedmont region (North–West Italy) by a consortium of 15 dairies following strict procedural guidelines. During the cheese manufacturing process, raw milk from two subsequent milking sessions (evening and morning) is inoculated with Natural Milk Cultures (NMCs), obtained by back-slopping process in which the previous days’ fermented material is inoculated into the fresh milk to start the production process. Then, the material is also inoculated with animal rennet and left to coagulate for 8–36 h at room temperature. Molding procedure takes place in perforated plastic containers for 24–48 h, where the cheeses are periodically turned over to allow the whey to release and manually dry salted in both sides (Gazzetta Ufficiale della Repubblica Italiana, 2013). Robiola di Roccaverano can be consumed as fresh cheese after 5 days from the beginning of production or ripened for 15 days. During the production of Robiola di Roccaverano, the cheesemakers naturally select the best performing NMC that are well adapted to the dairy environment, able to ensure reproducibility and standardization of the final product without losing its typicality. Therefore, the sensory characteristics of Robiola di Roccaverano are highly influenced by the action of the microorganism deriving from raw milk and NMC (Biolcati et al., 2019, 2020, 2021), as well as by contaminating microorganisms often deriving from sources such as operators, dairy equipment, and environment (Montel et al., 2014). Despite understudied, the fungal composition of the dairy products, commonly referred to as mycobiota, could strongly influence the composition of the final cheese and must be considered for a better characterization of PDO cheese.

In this work, the microbiome, both bacterial and fungal composition, of Robiola di Roccaverano has been studied by means of High Throughput Sequencing (HTS) approach. Cheese samples from different cheesemakers have been collected since the bacterial microbiota and the mycobiota may be associated with the geographical area, season, dairy environment, or raw materials used for the production. Nevertheless, due to the strict manufacture guidelines, it might be possible to define a common core microbial population, typical of Robiola di Roccaverano PDO cheese.

## Materials and Methods

### Samples Collection

Cheeses after 5 and 15 days of ripening were collected from five dairy plants (A, B, C, D, and E) located in different Roccaverano area of the Piedmont region. Six different batches were collected from each dairy plant (3 for 5-day and 15-day ripened cheese, respectively). For each batch, sampling took place on the same days and at the same time of year (January–February). All the producers used only goat’s milk for the production with the addition of NMC made by each cheesemaker. The 30 samples were collected aseptically and transported in laboratory at 4–6°C and kept at –20°C before the analysis.

### DNA Extraction

Ten grams of cheese were homogenized in a sterile stomacher bag with 90 milliliter of sterile Ringer solution (Oxoid, Milan, Italy) and mixed in a Stomacher 400 Circulator (Seward, United Kingdom) at 300 rpm for 3 min. Due to the soft texture of Robiola di Roccaverano cheese, no distinction between core and rind was made during sampling. One milliliter of the first decimal dilution was transferred into 1.5 ml micro-tube, and DNA was extracted by using the DNeasy Blood & Tissue kit (Qiagen, Hilden, Germany) following manufacturer’s instruction. DNA was extracted and quantified by using the QUBIT dsHS kit (Thermo Fisher, Milan, Italy), and purity was checked by absorbance measure using the spectrophotometer (Nanodrop 2000, Thermo Fisher Scientific, Waltham, MA, United States).

### High-Throughput Sequencing Analysis

The bacterial community was analyzed by amplifying the V3–V4 hypervariable region of the 16S rRNA gene using primers and procedure described by Klindworth et al. (2013). The fungal population was studied by amplification of the D1 domain of the 26S rRNA gene using primers and conditions described by Mota-Gutierrez et al. (2019). 26S rRNA target was chosen because it showed greater taxonomic resolution and robustness compared to the ITS region. PCR amplicons were purified following the Illumina metagenomic pipeline (Illumina Inc., San Diego, CA, United States). Sequencing was performed with a MiSeq platform (Illumina), generating 250 bp paired-end reads.

### Bioinformatic Analysis

Paired-end reads were imported in QIIME2 software for chimeric join and quality filtering (Caporaso et al., 2010; Edgar et al., 2011; Magoč and Salzberg, 2011; Bolyen et al., 2019). Amplicon sequence variants (ASVs) generated through DADA2 were used for taxonomic assignment against the Greengenes 16S rRNA gene database for bacteria and using the in-house database of Mota-Gutierrez et al. (2019) for fungi (Wang et al., 2007; Callahan et al., 2016). ASV taxonomy assignment was then confirmed by manual blast. The ASV table obtained with QIIME2 was rarefied at the lowest number of sequences, and when displayed, the higher taxonomy resolution was reached. When the taxonomy assignment was not able to reach species level, the genus or family name was displayed. PICRUSt (Langille et al., 2013) was used to predict the abundance of the KEGG gene families, based on 16S rRNA sequence data (Bokulich et al., 2015; Ferrocino et al., 2016). KEGG orthologs were then collapsed at a hierarchy level of 3, and the table was imported into R.

Sequencing data were deposited in the Sequence Read Archive of the National Center for Biotechnology Information (NCBI)^1^ under Bioproject accession numbers PRJNA704571 and PRJNA626588.

### Statistical Analysis

Alpha diversity indices were calculated with vegan package (Dixon, 2003) in R environment. ASV tables obtained through QIIME2 were imported in R^2^, using made4 function to obtain the Principal Component Analysis (PCA) plots. Venn diagrams were obtained by using Venn Diagram Maker. Inferred metagenome data were analyzed by the *made4* package, and hierarchical Ward-linkage clustering, based on the Spearman correlation coefficients of the proportion of the activities belonging to lipid carbohydrates and amino acid metabolism pathways, was used to produce a heatplot.

## Results

### Bacterial Microbiota Composition of Robiola di Roccaverano Cheese

**FIGURE 1:**
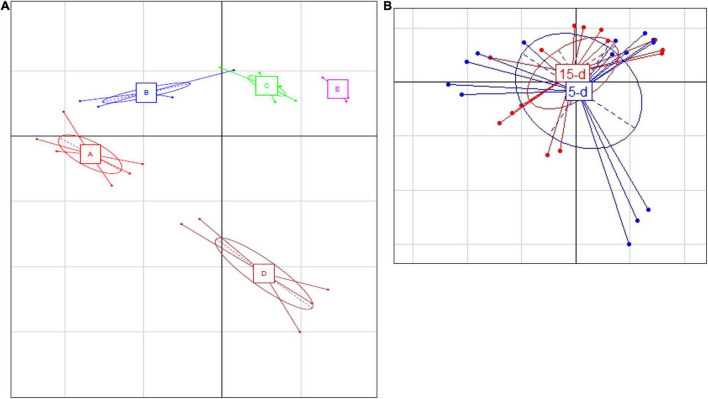
**(A)** Principal Component Analysis based on ASV relative abundance of bacterial microbiota targeted by 16S rRNA. **(A)** Differences between the different dairy plants; the first component (horizontal) accounts for the 59.91% of the variance, and the second component (vertical) accounts for the 10.84%. The five different plants are indicated as follows: A (red bars), B (blue bars), C (green bars), D (brown bars), and E (magenta bars). **(B)** Differences between 5-day (blue) and 15-day ripened cheese (red) analyzed; the first component (horizontal) accounts for the 28.16% of the variance, and the second component (vertical) accounts for the 21.85%. Ellipses in the graph are drown around similar cluster. The bars which originated from a common centroid highlighted the difference between samples belonging to the same cluster.

**FIGURE 2:**
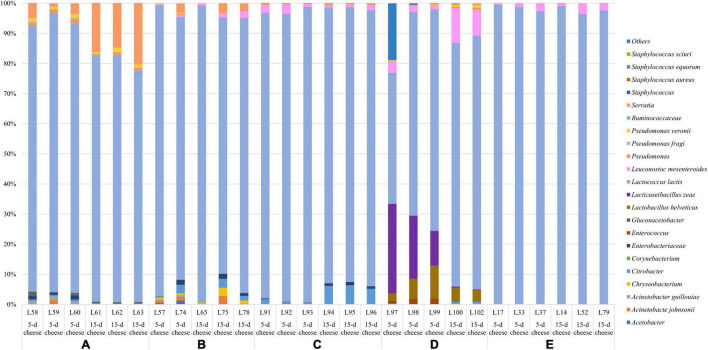
Relative abundance (%) of the taxa detected with 16S sequencing. Only ASVs with an incidence at least 0.5% in at least two samples are shown.

**FIGURE 3:**
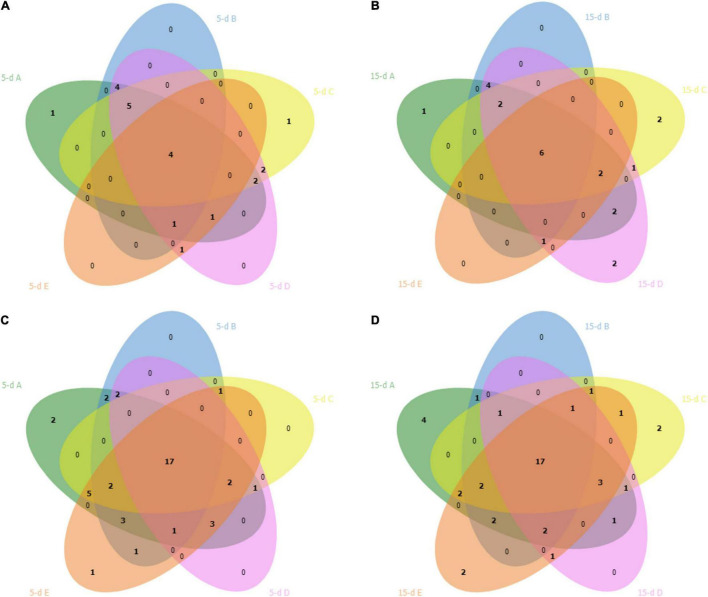
Venn diagram shows overlap of bacterial ASVs in cheese after 5 days (5-d) of ripening (plot **A**) and after 15 days (15-d) of ripening (plot **B**) across the five dairy plants. Plots **(C,D)** showed the overlap of fungal ASVs in cheese after 5 and 15 days of ripening, respectively. The dairy plants names are indicated with the letter A (green), B (blue), C (yellow), D (pink) and E (red).

After sequencing, two samples were excluded from 16S rRNA dataset (one sample from dairy B and one sample from dairy D) due to lower number of sequences. The PCA (Figure 1A) showed that cheese samples were grouped according to the dairy plants, and no separation was observed between 5- and 15-day ripened cheese collected from the same dairy plant (Figure 1B). ASVs with a relative abundance of at least 0.5% in two samples are shown in Figure 2 and Supplementary Table 1. ASVs shared across different cheese type and dairy plant were analyzed by a Venn diagram analysis in order to detect unique ASVs or shared ones (Figure 3). In samples after 5 days of ripening, *L. lactis*, *L. mesenteroides*, *Serratia* sp., and the family *Enterobacteriaceae* were common to all dairy plants. *Staphylococcus sciuri* was unique of dairy C, while *Ruminococcaceae* were observed only in dairy plant A. After 15 days of ripening, *Enterococcus* sp. and *Acinetobacter johnsonii* were shared in the dataset, while *Staphylococcus equorum* was detected only in plant A, while *Lactobacillus helveticus* and *Lactobacillus zeae* were found mainly in plant D. Finally, *S. sciuri* and *Ruminococcaceae* were found unique in plant C (Figures 2, 3 and Supplementary Table 1).

*Lactococcus lactis* was the observed species with the highest relative abundance across all the samples, although it displayed some statistical differences between plants. Dairy E exhibit the highest relative frequency of *L. lactis* (from 96% to > 99%), followed by dairy plant C (from 91 to 99%), A and B showed similar relative abundance (from 76 to 99%), and D had the lowest relative abundance (from 53 to 84%). *L. mesenteroides* was found in all cheese samples with a relative abundance which reached 11%. In addition, *Enterococcus* spp. was observed as subdominant genus across all the samples.

Other taxa detected belonged to psychrotrophic genera such as *Acinetobacter*, *Chryseobacterium*, *Corynebacterium*, *Pseudomonas*, and *Serratia.* Additionally, few ASVs belonging to *Staphylococcus* sp. and the undesirable species *S. aureus* were observed in some samples at 1% of the relative abundance. The inferred metagenome was depicted in the heatplot, and two main cluster were identified (Supplementary Figure 1). Cheese samples collected from dairy plant A and B belong to cluster 1, dairy plant E and C belong to the cluster 2, while dairy D was quite spread between the two group. No specific distribution of cheeses at different ripening time was then observed. The 5-day cheeses of the dairy plant E and C showed a higher abundance of KEGG genes related to the lipid metabolism such as fatty acid and sphingolipid metabolism, biosynthesis of unsaturated fatty acids, and KEGG genes involved in citrate cycle (Supplementary Figure 1).

### Mycobiota Composition of Robiola di Roccaverano Cheese

**FIGURE 4:**
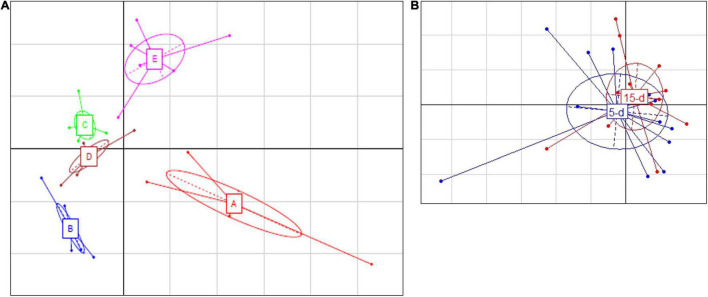
Principal Component Analysis based on ASV relative abundance of fungal microbiota targeted by 26S rRNA gene. **(A)** Differences among different dairy plants; the first component (horizontal) accounts for the 28.16% of the variance, and the second component (vertical) accounts for the 21.85%. The five different dairy plants are indicated as follows: A (red bars), B (blue bars), C (green bars), D (brown bars), and E (magenta bars). **(B)** Differences between 5-day (blue) and 15-day ripened cheese (red) analyzed. The first component (horizontal) accounts for the 20.20% of the variance, and the second component (vertical) accounts for the 15.03%. Ellipses in the graph are drown around similar cluster. The bars which originated from a common centroid highlighted the difference between samples belonging to the same cluster.

**FIGURE 5:**
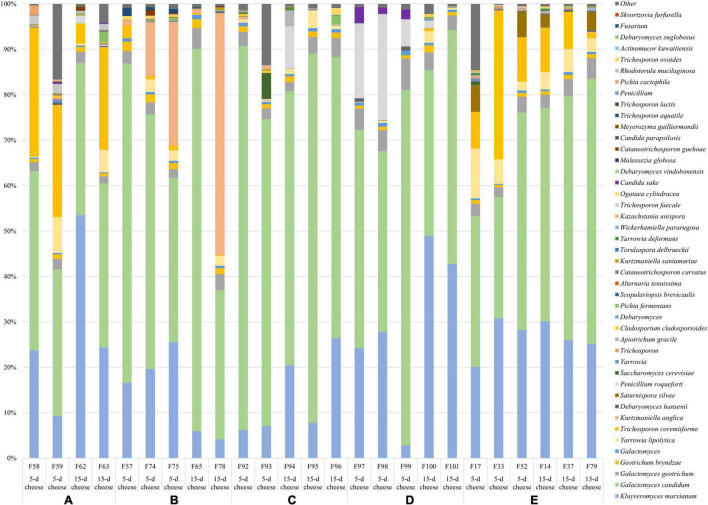
Relative abundance (%) of the taxa detected with 26S sequencing. Only ASVs with an incidence at least 0.5% in at least two samples are shown.

Five samples from the 26S rDNA sequencing were excluded from the analysis (two samples from dairy A; one sample from dairy B; one sample from dairy C; one sample from dairy D) due to poor DNA quality. The PCA analysis of the 26S data showed a cluster of cheese samples according to the plant (Figure 4A) independently of the time of ripening (Figure 4B). Venn diagram showed (Figure 3) 17 common ASVs in 5- and 15-day cheese. *Galactomyces candidum* and its telomorphic counterpart *Geotrichum candidum* (with a relative abundance accounting from 26 to 85%) and *Kluyveromyces marxianus* (with a relative abundance accounting from 4 to 49%) were the most abundant (Figure 5 and Supplementary Table 2). Several minor fraction of ASVs were in common in the dataset belonging to *Yarrowia lipolytica*, *Debaryomyces hansenii*, *Trichosporon coremiiforme*, *Penicillium roqueforti*, *Kurtzmaniella anglica*, *Pichia fermentans*, *Geotrichum bryndzae*, *Kurtzmaniella santamariae*, *Wickerhamiella pararugosa*, *Cladosporium cladosporioides*, *Trichosporon*, *Galactomyces*, and *Yarrowia* (for all details, see Supplementary Table 2). Some ASVs were detected in a specific dairy plant (Figures 3, 5). In 5-day ripened cheese, *Candida parapsilosis*, *Rhodotorula mucilaginosa*, *Candida sake*, and *Fusarium* spp. were detected in dairy A. Dairy plant C were characterized by the presence of *Meyerozyma guilliermondii* and *Skvortzovia furfurella*. Finally, some ASVs were found only in dairy plant E such as *Actinomucor kuwaitiensis* and *Debaryomyces vindobonensis* (Figures 3, 5). In 15-day ripened cheese, some differences have been observed. Dairy plant A was characterized by *R. mucilaginosa* and *S. furfurella*, while *Torulospora delbrueckii* (Figures 3, 5) was found only in dairy E.

## Discussion

The present study aimed to characterize bacterial and fungal population of Robiola di Roccaverano cheese collected from five dairy plants. *L. lactis* was the dominant ASVs in all cheeses collected from the different plants probably due to the addition of natural ferments during the manufacturing process (Wouters et al., 2002). *L. lactis* is an important component of the defined and undefined starters worldwide (Erkus et al., 2013) selected mainly for its ability to rapidly acidify milk during the cheese-making process. In addition to its starter activity, *L. lactis* has also been recognized for its ability to produce important aromatic compounds during cheese production (Ruggirello et al., 2018). In this study, samples from dairy plants E and C showed the highest frequency of *L. lactis* and the corresponding samples were mostly associated to inferred KEGG genes involved in lipid and citrate metabolism (Supplementary Figure 1). The principal compounds obtained through the citrate route are acetate, diacetyl, acetoin, and 2,3-butanediol, which has already been associated with *L. lactis* in miniaturized cheese (Ruggirello et al., 2018). In addition, it was reported that *L. lactis* displays the capability to metabolized carbohydrates through citrate route (McSweeney and Sousa, 2000). Strains belonging to the same species can have a different metabolic behavior; thus, we can hypothesize that the dairy environment selects specific strains that possess a strong lipolytic activity essential for cheese flavor development. The biological feature of the bacterial microbiota in this cheese can be due to the metabolic activity of specific *L. lactis* from dairy plant E and C. Among lactic acid bacteria, other ASVs were *L. mesenteroides*, *Lb. helveticus*, and *Lcb. zeae. Lb. helveticus* is commonly used as starter in Swiss-type cheese like Emmental but also to produce long-time ripened Italian cheeses like Provolone where its intense proteolytic activity is important during ripening process (Griffiths and Tellez, 2013). In contrast, the presence of *Lcb. zeae* has not been previously found in cheese made with NMC like Montasio and the so-called Traditional Specialty Guaranteed (TSG) Mozzarella cheese (Marino et al., 2003; Guidone et al., 2016).

Excluding plant E, which was probably more able to standardize the cheese production preventing the growth of undesirable species, the other dairies harbored several ASVs belonging to contaminant taxa. The presence of psychrotrophic bacteria like *Pseudomonas* spp., *Acinetobacter* spp., and *Enterobacteriaceae*, among which the genus *Serratia*, is common in raw milk microbiota (Ercolini et al., 2009). Gram-negative bacteria are commonly present in milk samples as they are linked to animal and environmental factors and induce spoilage of dairy foods (Hantsis-Zacharov et al., 2008; Ercolini et al., 2009; Montel et al., 2014). Although the results concerning the safety aspects could be considered promising, the viable bacterial flora was not evaluated in this study. The presence of toxin-producing S*taphylococcus aureus*, which is a common contaminant of raw milk and raw milk cheese, was not observed in Robiola di Roccaverano cheeses. Therefore, it could be postulated that the technology used in this study for the microbial characterization does not allow the detection of subdominant microbial population, among which S*taphylococcus aureus*.

Fungi possess an active role in the technological process related to the cheese production; however, they are also involved in spoilage phenomena. Indeed, some yeast species are recognized as primary drivers of defects during cheese making process, causing economic losses and leading to food safety issue (Kure and Skaar, 2019). Many studies have revealed that a great variety of fungal species are strictly associated with specific types of cheese, but few species tend to dominate the cheese systems (Marín et al., 2015; Anelli et al., 2019; Kure and Skaar, 2019). In the present study, the core mycobiota consisted mainly of *G. candidum* followed by *K. marxianus*, which were found in high abundance in all the samples. *G. candidum* has been found in various environmental sources like milk and soft cheeses and recently also in fermented milk like *Gioddu* and *Gwell* (Boutrou and Guéguen, 2005; Maoloni et al., 2020; von Gastrow et al., 2020). The yeast *K. marxianus* could be a component of whey starter culture and NMC because of its ability to ferment lactose through β-galactosidase activity. Recently, this species has been isolated from the typical hard-cheese *Fiore Sardo* (Fadda et al., 2017) where it influenced the ripening process through lactic acid utilization and by its proteolytic and lipolytic activity.

Other relevant yeasts observed in minor percentage were *Y. lipolytica*, *D. hansenii*, and *T. coremiiforme. Debaryomyces* spp. is frequently detected in milk and dairy products due to its resistance to low pH and high salt content (Ferrocino et al., 2020).

*Yarrowia lipolytica* represents one of the most common yeast species, but it cannot be added deliberately. In fact, it does not belong to the species commonly used to produce commercial starter culture used in the dairy industry. It is well known for its strong proteolytic and lipolytic activity which influence aroma and texture formation; thus, it is therefore particularly involved in ripening (Suzzi et al., 2001). This yeast has already been detected by HTS in short-ripened cheese like *Tomme d’Orchies* (Ceugniez et al., 2017).

Some detected ASVs were characteristic of specific dairy plant such as *P. roqueforti* in dairy D. This mold is often present in artisanal blue or white mold cheeses (Kure and Skaar, 2019). It is known to possess the ability to produce volatile aromatic compound due to its strong lipolytic and proteolytic activity, which could have a positive impact on flavor formation during blue-cheese production (Caron et al., 2021). Moreover, *Saturnispora silvae*, particularly detected in dairy E, was already isolated and identified from Robiola di Roccaverano cheese by culture-dependent methods and could be ascribed to an environmental source of contamination (Biolcati et al., 2020, 2021). Finally, *Kurtzmaniella anglica* and *Trichosporon coremiiforme*, which were found mainly in dairy plants A, B, and E, respectively, were already detected in a previous work on Robiola di Roccaverano cheese (Biolcati et al., 2020).

Some of the genera detected in low abundance in this study, namely, *Penicillium*, *Cladosporium*, and *Fusarium*, are undesirable fungi responsible for spoilage. Among them, *Penicillium* spp. constituted the largest group of spoilage molds in food and some species could be responsible for the production of mycotoxin (Garnier et al., 2017). *Cladosporium* spp. growing poorly is considered quite common in air system (Kure and Skaar, 2019). Mold contamination through ambient air is normal, more than for the yeast. Therefore, it is of notable importance, especially for small producers of artisanal cheeses such as Robiola di Roccaverano diaries, to follow accurate hygienic practice during the manufacturing and packaging to prevent the contamination.

## Conclusion

In this study, metataxonomic analysis allowed the characterization of the microbial and fungal population of cheese samples collected from different Robiola di Roccaverano diaries. The differences detected suggested that, despite the localization of the dairy plants in different areas and the raw materials used, i.e., raw goat’s milk and NMC, it is possible to define a core microbial population constituted by *L. lactis*, *G. candidum*, and *K. marxianus*. However, some dairy-specific microbial and fungal traits have been identified and may help in the definition of specific hallmark.

## Data Availability Statement

Sequencing data were deposited in the Sequence Read Archive of the National Center for Biotechnology Information (NCBI; https://www.ncbi.nlm.nih.gov/) under Bioproject accession numbers PRJNA704571 and PRJNA626588.

## Author Contributions

FB: sample collection and preparation, and manuscript drafting. FB and IF: sequencing, data analysis, and interpretation. MB and AD: project administration, conceptualization, study design, and funding acquisition. All authors contributed to manuscript revision and read and approved the submitted version.

## Conflict of Interest

The authors declare that the research was conducted in the absence of any commercial or financial relationships that could be construed as a potential conflict of interest.

## Publisher’s Note

All claims expressed in this article are solely those of the authors and do not necessarily represent those of their affiliated organizations, or those of the publisher, the editors and the reviewers. Any product that may be evaluated in this article, or claim that may be made by its manufacturer, is not guaranteed or endorsed by the publisher.

## Footnotes

1
https://www.ncbi.nlm.nih.gov/

2
www.r-project.org

## Supplementary Material

Supplementary Figure 1 Heatplot of the abundance of KEGG gene belonging to carbohydrates, amino acid, and lipid metabolism pathways (left bar) in 5- and 15-day ripened cheese (upper bar from the top) and in the different dairy plant (lower bar from the top). The intensity of the color represents the degree of correlation between samples and inferred metabolic pathways.
