# Supplementary figures and images for: The Bacterial and Fungal Microbiota of “Robiola di Roccaverano” Protected Designation of Origin Raw Milk Cheese
Source: Front Microbiol. 2022 Jan 31;12:776862. doi: 10.3389/fmicb.2021.776862 (PMC8841559; doi:10.3389/fmicb.2021.776862)

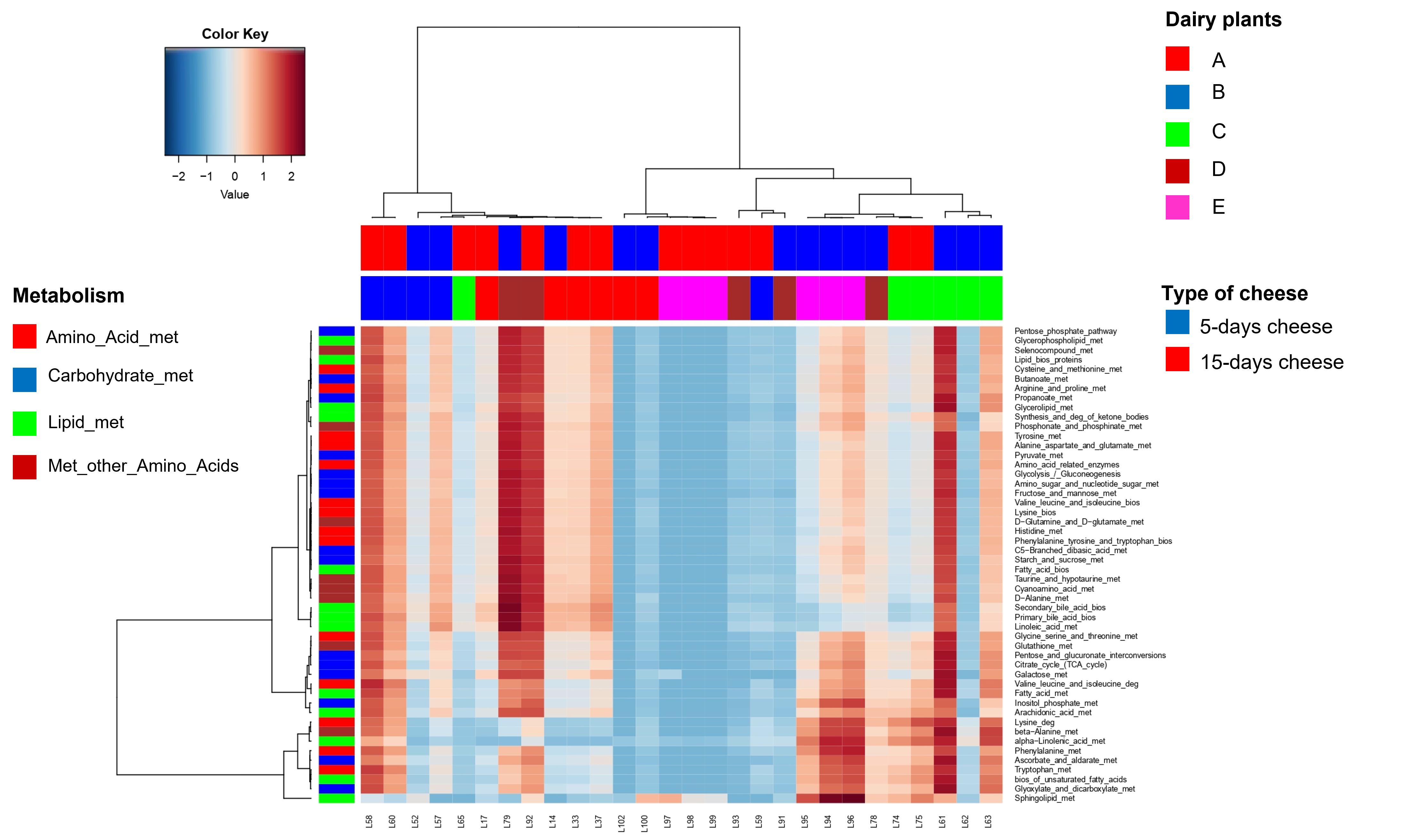

Supplement: Supplementary Figure 1 — Heatplot of the abundance of KEGG gene belonging to carbohydrates, amino acid, and lipid metabolism pathways (left bar) in 5- and 15-day ripened cheese (upper bar from the top) and in the different dairy plant (lower bar from the top). The intensity of the color represents the degree of correlation between samples and inferred metabolic pathways. [file Image_1.TIFF]
