# Supplementary material for: The Bacterial and Fungal Microbiota of “Robiola di Roccaverano” Protected Designation of Origin Raw Milk Cheese
Source: Front Microbiol. 2022 Jan 31;12:776862. doi: 10.3389/fmicb.2021.776862 (PMC8841559; doi:10.3389/fmicb.2021.776862)
Supplement: Supplementary file 2 [file Table_1.DOCX]

**Supplementary Table 1.** Relative abundance (%) of the taxa detected with 16S sequencing.

|  | Dairy Plant | A | A | A | A | A | A |
| --- | --- | --- | --- | --- | --- | --- | --- |
|  | Code | L58 | L59 | L60 | L61 | L62 | L63 |
|  | Matrix | 5-d cheese | 5-d cheese | 5-d cheese | 15-d cheese | 15-d cheese | 15-d cheese |
| *Proteobacteria* | *Acetobacter* | 0.00 | 0.01 | 0.00 | 0.00 | 0.00 | 0.00 |
|  | *Acinetobacte johnsonii* | 0.26 | 1.26 | 0.17 | 0.07 | 0.08 | 0.05 |
|  | *Acinetobacter guillouiae* | 0.63 | 1.22 | 0.74 | 0.20 | 0.08 | 0.09 |
|  | *Citrobacter* | 0.69 | 0.52 | 0.66 | 0.07 | 0.05 | 0.09 |
|  | *Enterobacteriaceae* | 1.16 | 0.76 | 1.30 | 0.01 | 0.01 | 0.01 |
|  | *Gluconacetobacter* | 1.44 | 0.07 | 0.87 | 0.50 | 0.46 | 0.49 |
|  | *Pseudomonas* | 0.85 | 1.12 | 1.20 | 0.35 | 0.68 | 0.67 |
|  | *Pseudomonas fragi* | 0.55 | 0.01 | 0.46 | 0.03 | 0.03 | 0.02 |
|  | *Pseudomonas veronii* | 1.09 | 0.85 | 1.32 | 0.56 | 1.60 | 1.15 |
|  | *Ruminococcaceae* | 0.00 | 0.01 | 0.00 | 0.00 | 0.00 | 0.00 |
|  | *Serratia* | 4.88 | 1.07 | 3.57 | 16.10 | 14.61 | 20.23 |
| *Bacteroidetes* | *Chryseobacterium* | 0.05 | 0.07 | 0.00 | 0.01 | 0.01 | 0.03 |
| *Actinobacteria* | *Corynebacterium* | 0.00 | 0.00 | 0.01 | 0.00 | 0.01 | 0.01 |
| *Firmicutes* | *Enterococcus* | 0.00 | 0.06 | 0.02 | 0.00 | 0.02 | 0.00 |
|  | *Lactobacillus helveticus* | 0.00 | 0.00 | 0.00 | 0.00 | 0.00 | 0.00 |
|  | *Lacticaseibacillus zeae* | 0.00 | 0.01 | 0.00 | 0.00 | 0.00 | 0.00 |
|  | *Lactococcus lactis* | 87.77 | 90.65 | 89.18 | 81.60 | 81.88 | 76.73 |
|  | *Leuconostoc mesenteroides* | 0.01 | 0.00 | 0.03 | 0.11 | 0.07 | 0.12 |
|  | *Staphylococcus* | 0.01 | 0.00 | 0.00 | 0.02 | 0.01 | 0.01 |
|  | *Staphylococcus aureus* | 0.00 | 0.00 | 0.00 | 0.01 | 0.00 | 0.00 |
|  | *Staphylococcus equorum* | 0.00 | 0.00 | 0.00 | 0.00 | 0.01 | 0.00 |
|  | *Staphylococcus sciuri* | 0.00 | 0.00 | 0.00 | 0.00 | 0.00 | 0.00 |

|  | Dairy Plant | B | B | B | B | B |
| --- | --- | --- | --- | --- | --- | --- |
|  | Code | L57 | L65 | L74 | L75 | L78 |
|  | Matrix | 5-d cheese | 15-d cheese | 5-d cheese | 15-d cheese | 5-d cheese |
| *Proteobacteria* | *Acetobacter* | 0.46 | 0.04 | 1.26 | 0.11 | 0.07 |
|  | *Acinetobacte johnsonii* | 0.70 | 0.07 | 1.21 | 2.55 | 0.13 |
|  | *Acinetobacter guillouiae* | 0.50 | 0.22 | 0.52 | 0.17 | 0.01 |
|  | *Citrobacter* | 0.10 | 0.13 | 2.84 | 2.97 | 1.52 |
|  | *Enterobacteriaceae* | 0.08 | 0.04 | 1.61 | 1.62 | 0.88 |
|  | *Gluconacetobacter* | 0.13 | 0.00 | 0.10 | 0.07 | 0.05 |
|  | *Pseudomonas* | 0.01 | 0.00 | 0.33 | 0.31 | 0.32 |
|  | *Pseudomonas fragi* | 0.01 | 0.00 | 0.00 | 0.00 | 0.00 |
|  | *Pseudomonas veronii* | 0.12 | 0.01 | 0.28 | 0.12 | 0.01 |
|  | *Ruminococcaceae* | 0.00 | 0.00 | 0.00 | 0.00 | 0.00 |
|  | *Serratia* | 0.10 | 0.07 | 3.12 | 2.91 | 2.15 |
| *Bacteroidetes* | *Chryseobacterium* | 0.61 | 0.37 | 0.63 | 2.61 | 1.05 |
| *Actinobacteria* | *Corynebacterium* | 0.00 | 0.00 | 0.00 | 0.00 | 0.00 |
| *Firmicutes* | *Enterococcus* | 0.02 | 0.00 | 0.02 | 0.02 | 0.01 |
|  | *Lactobacillus helveticus* | 0.00 | 0.00 | 0.00 | 0.00 | 0.00 |
|  | *Lacticaseibacillus zeae* | 0.00 | 0.00 | 0.00 | 0.00 | 0.00 |
|  | *Lactococcus lactis* | 96.30 | 98.32 | 86.70 | 84.30 | 90.75 |
|  | *Leuconostoc mesenteroides* | 0.39 | 0.68 | 0.78 | 1.35 | 2.42 |
|  | *Staphylococcus* | 0.00 | 0.00 | 0.00 | 0.00 | 0.00 |
|  | *Staphylococcus aureus* | 0.00 | 0.00 | 0.00 | 0.00 | 0.00 |
|  | *Staphylococcus equorum* | 0.00 | 0.00 | 0.00 | 0.00 | 0.00 |
|  | *Staphylococcus sciuri* | 0.00 | 0.00 | 0.00 | 0.00 | 0.00 |

|  | Dairy Plant | C | C | C | C | C | C |
| --- | --- | --- | --- | --- | --- | --- | --- |
|  | Code | L91 | L92 | L93 | L94 | L95 | L96 |
|  | Matrix | 5-d cheese | 5-d cheese | 5-d cheese | 15-d cheese | 15-d cheese | 15-d cheese |
| *Proteobacteria* | *Acetobacter* | 0.00 | 0.00 | 0.00 | 0.00 | 0.00 | 0.00 |
|  | *Acinetobacte johnsonii* | 0.08 | 0.04 | 0.04 | 0.09 | 0.01 | 0.05 |
|  | *Acinetobacter guillouiae* | 0.07 | 0.02 | 0.02 | 0.06 | 0.02 | 0.01 |
|  | *Citrobacter* | 1.55 | 0.71 | 0.64 | 5.94 | 6.31 | 5.15 |
|  | *Enterobacteriaceae* | 0.25 | 0.11 | 0.08 | 0.89 | 1.10 | 0.80 |
|  | *Gluconacetobacter* | 0.00 | 0.00 | 0.00 | 0.00 | 0.00 | 0.00 |
|  | *Pseudomonas* | 0.01 | 0.00 | 0.00 | 0.00 | 0.00 | 0.00 |
|  | *Pseudomonas fragi* | 0.00 | 0.00 | 0.00 | 0.00 | 0.00 | 0.00 |
|  | *Pseudomonas veronii* | 0.00 | 0.00 | 0.01 | 0.00 | 0.00 | 0.00 |
|  | *Ruminococcaceae* | 0.00 | 0.00 | 0.00 | 0.01 | 0.00 | 0.00 |
|  | *Serratia* | 0.33 | 0.22 | 0.18 | 0.69 | 0.33 | 0.55 |
| *Bacteroidetes* | *Chryseobacterium* | 0.00 | 0.00 | 0.00 | 0.00 | 0.00 | 0.00 |
| *Actinobacteria* | *Corynebacterium* | 0.02 | 0.01 | 0.01 | 0.00 | 0.00 | 0.00 |
| *Firmicutes* | *Enterococcus* | 0.00 | 0.00 | 0.00 | 0.00 | 0.00 | 0.01 |
|  | *Lactobacillus helveticus* | 0.00 | 0.00 | 0.00 | 0.00 | 0.00 | 0.00 |
|  | *Lacticaseibacillus zeae* | 0.00 | 0.00 | 0.00 | 0.00 | 0.00 | 0.00 |
|  | *Lactococcus lactis* | 94.73 | 95.60 | 98.01 | 91.49 | 91.13 | 91.70 |
|  | *Leuconostoc mesenteroides* | 2.71 | 3.18 | 0.94 | 0.73 | 1.00 | 1.65 |
|  | *Staphylococcus* | 0.02 | 0.03 | 0.01 | 0.01 | 0.00 | 0.00 |
|  | *Staphylococcus aureus* | 0.01 | 0.01 | 0.00 | 0.00 | 0.01 | 0.00 |
|  | *Staphylococcus equorum* | 0.00 | 0.00 | 0.00 | 0.00 | 0.00 | 0.00 |
|  | *Staphylococcus sciuri* | 0.11 | 0.03 | 0.04 | 0.00 | 0.00 | 0.01 |

|  | Dairy Plant | D | D | D | D | D |
| --- | --- | --- | --- | --- | --- | --- |
|  | Code | L97 | L98 | L99 | L100 | L102 |
|  | Matrix | 5-d cheese | 5-d cheese | 5-d cheese | 15-d cheese | 15-d cheese |
| *Proteobacteria* | *Acetobacter* | 0.00 | 0.01 | 0.00 | 0.01 | 0.00 |
|  | *Acinetobacte johnsonii* | 0.00 | 0.00 | 0.01 | 0.13 | 0.13 |
|  | *Acinetobacter guillouiae* | 0.01 | 0.00 | 0.00 | 0.03 | 0.04 |
|  | *Citrobacter* | 0.28 | 0.07 | 0.00 | 0.63 | 0.69 |
|  | *Enterobacteriaceae* | 0.00 | 0.01 | 0.00 | 0.01 | 0.00 |
|  | *Gluconacetobacter* | 0.02 | 0.01 | 0.00 | 0.30 | 0.21 |
|  | *Pseudomonas* | 0.06 | 0.04 | 0.10 | 0.41 | 0.60 |
|  | *Pseudomonas fragi* | 0.01 | 0.00 | 0.00 | 0.04 | 0.14 |
|  | *Pseudomonas veronii* | 0.12 | 0.14 | 0.43 | 0.59 | 0.77 |
|  | *Ruminococcaceae* | 0.00 | 0.00 | 0.00 | 0.00 | 0.00 |
|  | *Serratia* | 0.43 | 0.21 | 0.36 | 0.53 | 0.60 |
| *Bacteroidetes* | *Chryseobacterium* | 0.07 | 0.02 | 0.05 | 0.05 | 0.04 |
| *Actinobacteria* | *Corynebacterium* | 0.01 | 0.00 | 0.02 | 0.00 | 0.02 |
| *Firmicutes* | *Enterococcus* | 1.00 | 1.64 | 1.88 | 0.00 | 0.02 |
|  | *Lactobacillus helveticus* | 3.11 | 6.79 | 10.88 | 4.29 | 3.52 |
|  | *Lacticaseibacillus zeae* | 36.59 | 21.06 | 11.49 | 0.34 | 0.24 |
|  | *Lactococcus lactis* | 53.51 | 67.87 | 73.48 | 79.62 | 83.23 |
|  | *Leuconostoc mesenteroides* | 4.70 | 2.02 | 1.07 | 11.41 | 8.45 |
|  | *Staphylococcus* | 0.00 | 0.00 | 0.05 | 0.04 | 0.00 |
|  | *Staphylococcus aureus* | 0.00 | 0.02 | 0.02 | 0.01 | 0.00 |
|  | *Staphylococcus equorum* | 0.00 | 0.00 | 0.00 | 0.00 | 0.00 |
|  | *Staphylococcus sciuri* | 0.00 | 0.00 | 0.00 | 0.00 | 0.00 |

|  | *Dairy Plant* | E | E | E | E | E | E |
| --- | --- | --- | --- | --- | --- | --- | --- |
|  | *CODE* | L52 | L17 | L33 | L14 | L37 | L79 |
|  | *Matrix* | 15-d cheese | 5-d cheese | 5-d cheese | 15-d cheese | 5-d cheese | 15-d cheese |
| *Proteobacteria* | *Acetobacter* | 0.01 | 0.00 | 0.00 | 0.00 | 0.01 | 0.00 |
|  | *Acinetobacte johnsonii* | 0.00 | 0.00 | 0.00 | 0.01 | 0.00 | 0.00 |
|  | *Acinetobacter guillouiae* | 0.00 | 0.00 | 0.00 | 0.00 | 0.00 | 0.00 |
|  | *Citrobacter* | 0.00 | 0.00 | 0.00 | 0.00 | 0.00 | 0.00 |
|  | *Enterobacteriaceae* | 0.00 | 0.01 | 0.00 | 0.00 | 0.00 | 0.01 |
|  | *Gluconacetobacter* | 0.00 | 0.00 | 0.00 | 0.00 | 0.00 | 0.00 |
|  | *Pseudomonas* | 0.00 | 0.00 | 0.00 | 0.00 | 0.00 | 0.00 |
|  | *Pseudomonas fragi* | 0.00 | 0.00 | 0.00 | 0.00 | 0.00 | 0.00 |
|  | *Pseudomonas veronii* | 0.00 | 0.00 | 0.00 | 0.00 | 0.00 | 0.00 |
|  | *Ruminococcaceae* | 0.00 | 0.00 | 0.00 | 0.00 | 0.00 | 0.00 |
|  | *Serratia* | 0.00 | 0.03 | 0.02 | 0.00 | 0.03 | 0.01 |
| *Bacteroidetes* | *Chryseobacterium* | 0.00 | 0.00 | 0.00 | 0.00 | 0.00 | 0.00 |
| *Actinobacteria* | *Corynebacterium* | 0.00 | 0.00 | 0.00 | 0.00 | 0.00 | 0.00 |
| *Firmicutes* | *Enterococcus* | 0.09 | 0.00 | 0.00 | 0.05 | 0.00 | 0.00 |
|  | *Lactobacillus helveticus* | 0.00 | 0.00 | 0.01 | 0.00 | 0.00 | 0.00 |
|  | *Lacticaseibacillus zeae* | 0.00 | 0.00 | 0.00 | 0.00 | 0.02 | 0.00 |
|  | *Lactococcus lactis* | 96.30 | 99.54 | 98.69 | 99.02 | 97.40 | 97.63 |
|  | *Leuconostoc mesenteroides* | 3.58 | 0.37 | 1.27 | 0.89 | 2.51 | 2.32 |
|  | *Staphylococcus* | 0.00 | 0.00 | 0.00 | 0.00 | 0.00 | 0.01 |
|  | *Staphylococcus aureus* | 0.00 | 0.00 | 0.00 | 0.01 | 0.00 | 0.00 |
|  | *Staphylococcus equorum* | 0.00 | 0.00 | 0.00 | 0.00 | 0.00 | 0.00 |
|  | *Staphylococcus sciuri* | 0.00 | 0.00 | 0.00 | 0.00 | 0.00 | 0.00 |
