# Supplementary material for: The Bacterial and Fungal Microbiota of “Robiola di Roccaverano” Protected Designation of Origin Raw Milk Cheese
Source: Front Microbiol. 2022 Jan 31;12:776862. doi: 10.3389/fmicb.2021.776862 (PMC8841559; doi:10.3389/fmicb.2021.776862)
Supplement: Supplementary file 3 [file Table_2.DOCX]

**Supplementary Table 2.** Relative abundance (%) of the taxa detected with 26S sequencing.

| Dairy Plant | A | A | A | A | B | B | B | B | B |
| --- | --- | --- | --- | --- | --- | --- | --- | --- | --- |
| Code | F63 | F62 | F59 | F58 | F65 | F74 | F78 | F75 | F57 |
| Matrix | 15-d cheese | 15-d cheese | 5-d cheese | 5-d cheese | 15-d cheese | 5-d cheese | 15-d cheese | 5-d cheese | 5-d cheese |
| *Kluyveromyces marxianus* | 24.27 | 53.26 | 9.06 | 23.65 | 5.96 | 19.56 | 4.13 | 25.54 | 16.46 |
| *Galactomyces candidum* | 35.94 | 33.32 | 31.32 | 39.39 | 84.09 | 56.01 | 32.86 | 36.08 | 69.80 |
| *Galactomyces geotrichum* | 1.58 | 2.50 | 2.18 | 1.97 | 4.56 | 2.67 | 3.48 | 1.87 | 2.65 |
| *Geotrichum bryndzae* | 0.65 | 1.02 | 0.98 | 0.64 | 1.91 | 1.73 | 1.34 | 1.39 | 2.11 |
| *Galactomyces* | 0.23 | 0.37 | 0.28 | 0.34 | 1.00 | 0.53 | 0.52 | 0.45 | 0.68 |
| *Yarrowia lipolytica* | 4.89 | 0.39 | 7.67 | 0.18 | 0.02 | 2.77 | 2.17 | 2.34 | 0.02 |
| *Other* | 4.15 | 0.60 | 15.99 | 0.34 | 0.78 | 0.73 | 0.46 | 1.16 | 0.87 |
| *Trichosporon coremiiforme* | 22.39 | 4.42 | 23.97 | 28.34 | 0.43 | 0.71 | 0.17 | 1.13 | 2.85 |
| *Kurtzmaniella anglica* | 0.14 | 0.04 | 0.02 | 0.01 | 0.72 | 11.86 | 53.20 | 27.13 | 1.01 |
| *Debaryomyces hansenii* | 0.24 | 0.02 | 0.45 | 0.10 | 0.00 | 0.02 | 0.30 | 0.12 | 0.00 |
| *Saturnispora silvae* | 0.01 | 0.00 | 0.00 | 0.00 | 0.01 | 0.01 | 0.00 | 0.00 | 0.00 |
| *Penicillium roqueforti* | 0.25 | 1.67 | 0.02 | 0.14 | 0.00 | 0.00 | 0.00 | 0.00 | 0.00 |
| *Saccharomyces cerevisiae* | 0.00 | 0.00 | 0.00 | 0.00 | 0.00 | 0.00 | 0.01 | 0.00 | 0.00 |
| *Yarrowia* | 0.14 | 0.01 | 0.69 | 0.01 | 0.00 | 0.11 | 0.12 | 0.06 | 0.00 |
| *Trichosporon* | 0.27 | 0.12 | 0.32 | 0.27 | 0.02 | 0.00 | 0.00 | 0.01 | 0.02 |
| *Apiotrichum gracile* | 0.00 | 0.01 | 0.00 | 0.00 | 0.00 | 0.00 | 0.00 | 0.00 | 0.00 |
| *Cladosporium cladosporioides* | 0.03 | 0.00 | 0.45 | 0.00 | 0.00 | 0.00 | 0.01 | 0.00 | 0.01 |
| *Debaryomyces* | 0.01 | 0.00 | 0.02 | 0.00 | 0.00 | 0.02 | 0.02 | 0.04 | 0.00 |
| *Pichia fermentans* | 2.42 | 0.47 | 0.10 | 0.14 | 0.07 | 0.26 | 0.21 | 0.57 | 0.08 |
| *Scopulariopsis brevicaulis* | 0.00 | 0.00 | 0.00 | 0.00 | 0.00 | 0.00 | 0.00 | 0.00 | 0.00 |
| *Alternaria tenuissima* | 0.02 | 0.00 | 0.18 | 0.00 | 0.00 | 0.00 | 0.00 | 0.00 | 0.00 |
| *Cutaneotrichosporon curvatus* | 0.41 | 0.03 | 0.21 | 0.08 | 0.01 | 0.13 | 0.10 | 0.09 | 0.02 |
| *Kurtzmaniella santamariae* | 0.01 | 0.01 | 0.02 | 0.00 | 0.00 | 0.10 | 0.43 | 0.19 | 0.02 |
| *Torulaspora delbrueckii* | 0.00 | 0.00 | 0.00 | 0.00 | 0.00 | 0.00 | 0.00 | 0.00 | 0.00 |
| *Yarrowia deformans* | 0.00 | 0.00 | 0.00 | 0.01 | 0.00 | 0.00 | 0.00 | 0.00 | 0.00 |
| *Wickerhamiella pararugosa* | 0.00 | 0.00 | 0.00 | 0.00 | 0.00 | 0.01 | 0.02 | 0.01 | 0.01 |
| *Kazachstania unispora* | 0.00 | 0.00 | 0.01 | 0.00 | 0.01 | 0.26 | 0.00 | 0.01 | 0.09 |
| *Trichosporon faecale* | 1.26 | 0.38 | 1.98 | 1.92 | 0.01 | 0.00 | 0.00 | 0.00 | 0.01 |
| *Ogataea cylindracea* | 0.00 | 0.00 | 0.00 | 0.00 | 0.07 | 0.53 | 0.02 | 0.50 | 0.75 |
| *Candida sake* | 0.22 | 0.07 | 0.31 | 0.01 | 0.00 | 0.00 | 0.00 | 0.00 | 0.00 |
| *Debaryomyces vindobonensis* | 0.00 | 0.00 | 0.01 | 0.00 | 0.00 | 0.00 | 0.00 | 0.00 | 0.00 |
| *Malassezia globosa* | 0.00 | 0.00 | 0.02 | 0.00 | 0.00 | 0.00 | 0.00 | 0.00 | 0.00 |
| *Cutaneotrichosporon guehoae* | 0.00 | 0.77 | 0.00 | 0.00 | 0.00 | 1.25 | 0.22 | 0.29 | 0.00 |
| *Candida parapsilosis* | 0.00 | 0.09 | 0.15 | 0.04 | 0.00 | 0.00 | 0.00 | 0.00 | 0.00 |
| *Meyerozyma guilliermondii* | 0.00 | 0.00 | 0.00 | 0.00 | 0.00 | 0.00 | 0.00 | 0.00 | 0.00 |
| *Trichosporon aquatile* | 0.01 | 0.00 | 0.02 | 0.01 | 0.25 | 0.60 | 0.12 | 0.87 | 1.77 |
| *Trichosporon lactis* | 0.00 | 0.00 | 0.03 | 0.01 | 0.00 | 0.00 | 0.00 | 0.00 | 0.03 |
| *Penicillium* | 0.00 | 0.00 | 0.03 | 0.01 | 0.00 | 0.00 | 0.00 | 0.00 | 0.00 |
| *Pichia cactophila* | 0.03 | 0.00 | 0.41 | 2.11 | 0.00 | 0.00 | 0.00 | 0.00 | 0.00 |
| *Rhodotorula mucilaginosa* | 0.00 | 0.00 | 0.00 | 0.00 | 0.00 | 0.00 | 0.00 | 0.00 | 0.00 |
| *Trichosporon ovoides* | 0.00 | 0.00 | 0.00 | 0.00 | 0.00 | 0.00 | 0.00 | 0.00 | 0.00 |
| *Candida davisiana* | 0.00 | 0.00 | 0.00 | 0.00 | 0.00 | 0.00 | 0.00 | 0.00 | 0.00 |
| *Actinomucor kuwaitiensis* | 0.00 | 0.00 | 0.00 | 0.00 | 0.00 | 0.00 | 0.00 | 0.00 | 0.00 |
| *Debaryomyces suglobosus* | 0.00 | 0.00 | 0.01 | 0.00 | 0.00 | 0.00 | 0.00 | 0.00 | 0.00 |
| *Fusarium* | 0.00 | 0.00 | 0.12 | 0.00 | 0.00 | 0.00 | 0.00 | 0.00 | 0.00 |
| *Skvortzovia furfurella* | 0.00 | 0.00 | 0.02 | 0.00 | 0.00 | 0.00 | 0.00 | 0.00 | 0.00 |

| Dairy Plant | C | C | C | C | C | D | D | D | D | D |
| --- | --- | --- | --- | --- | --- | --- | --- | --- | --- | --- |
| Code | F95 | F94 | F93 | F92 | F96 | F100 | F97 | F98 | F101 | F99 |
| Matrix | 15-d cheese | 15-d cheese | 5-d cheese | 5-d cheese | 15-d cheese | 15-d cheese | 5-d cheese | 5-d cheese | 15-d cheese | 5-d cheese |
| *Kluyveromyces marxianus* | 7.75 | 20.49 | 6.99 | 6.15 | 26.40 | 48.77 | 24.21 | 27.83 | 42.75 | 2.82 |
| *Galactomyces candidum* | 81.24 | 60.22 | 66.92 | 83.90 | 61.86 | 36.33 | 48.03 | 39.69 | 51.38 | 78.21 |
| *Galactomyces geotrichum* | 3.68 | 2.02 | 2.31 | 3.10 | 4.14 | 3.88 | 4.70 | 4.68 | 3.25 | 6.89 |
| *Geotrichum bryndzae* | 1.21 | 1.27 | 0.90 | 1.26 | 1.19 | 1.56 | 0.63 | 0.73 | 0.80 | 0.70 |
| *Galactomyces* | 0.70 | 0.49 | 0.53 | 0.77 | 0.73 | 0.54 | 0.73 | 0.83 | 0.47 | 1.05 |
| *Yarrowia lipolytica* | 3.84 | 1.12 | 0.19 | 0.70 | 0.72 | 2.51 | 0.08 | 0.43 | 0.41 | 0.02 |
| *Other* | 0.99 | 0.58 | 13.30 | 1.38 | 0.90 | 2.21 | 0.67 | 0.70 | 0.57 | 1.17 |
| *Trichosporon coremiiforme* | 0.00 | 0.00 | 0.02 | 0.00 | 0.01 | 0.61 | 0.02 | 0.02 | 0.03 | 0.00 |
| *Kurtzmaniella anglica* | 0.01 | 0.05 | 0.18 | 0.62 | 0.03 | 0.00 | 0.02 | 0.02 | 0.01 | 0.00 |
| *Debaryomyces hansenii* | 0.02 | 0.02 | 0.02 | 0.02 | 0.01 | 0.03 | 0.75 | 0.10 | 0.02 | 0.84 |
| *Saturnispora silvae* | 0.00 | 0.01 | 0.00 | 0.00 | 0.01 | 0.00 | 0.00 | 0.01 | 0.00 | 0.00 |
| *Penicillium roqueforti* | 0.10 | 9.34 | 0.20 | 0.01 | 0.19 | 1.75 | 16.44 | 23.28 | 0.01 | 5.97 |
| *Saccharomyces cerevisiae* | 0.02 | 0.00 | 5.66 | 0.03 | 0.02 | 0.00 | 0.02 | 0.00 | 0.00 | 0.00 |
| *Yarrowia* | 0.18 | 0.05 | 0.00 | 0.01 | 0.05 | 0.27 | 0.00 | 0.04 | 0.01 | 0.00 |
| *Trichosporon* | 0.00 | 0.00 | 0.00 | 0.00 | 0.00 | 0.02 | 0.00 | 0.00 | 0.00 | 0.00 |
| *Apiotrichum gracile* | 0.00 | 3.40 | 0.00 | 0.00 | 0.00 | 0.00 | 0.00 | 0.00 | 0.00 | 0.00 |
| *Cladosporium cladosporioides* | 0.00 | 0.00 | 0.54 | 0.46 | 0.03 | 0.00 | 0.00 | 0.00 | 0.01 | 0.02 |
| *Debaryomyces* | 0.00 | 0.00 | 0.00 | 0.00 | 0.00 | 0.00 | 0.02 | 0.00 | 0.00 | 0.02 |
| *Pichia fermentans* | 0.00 | 0.00 | 0.00 | 0.00 | 1.92 | 0.28 | 0.02 | 0.03 | 0.06 | 0.00 |
| *Scopulariopsis brevicaulis* | 0.00 | 0.00 | 0.00 | 0.00 | 0.00 | 0.00 | 0.00 | 0.00 | 0.02 | 0.00 |
| *Alternaria tenuissima* | 0.00 | 0.00 | 0.05 | 0.04 | 0.01 | 0.00 | 0.00 | 0.00 | 0.00 | 0.00 |
| *Cutaneotrichosporon curvatus* | 0.00 | 0.00 | 0.00 | 0.04 | 0.03 | 0.07 | 0.00 | 0.00 | 0.00 | 0.00 |
| *Kurtzmaniella santamariae* | 0.01 | 0.00 | 0.02 | 0.00 | 0.00 | 0.00 | 0.00 | 0.00 | 0.01 | 0.00 |
| *Torulaspora delbrueckii* | 0.00 | 0.00 | 0.00 | 0.00 | 0.00 | 0.00 | 0.00 | 0.00 | 0.00 | 0.00 |
| *Yarrowia deformans* | 0.02 | 0.73 | 0.10 | 0.36 | 0.08 | 0.00 | 0.00 | 0.00 | 0.00 | 0.00 |
| *Wickerhamiella pararugosa* | 0.00 | 0.00 | 0.00 | 0.18 | 0.02 | 0.00 | 0.00 | 0.00 | 0.02 | 0.00 |
| *Kazachstania unispora* | 0.09 | 0.00 | 0.95 | 0.18 | 0.02 | 0.00 | 0.00 | 0.03 | 0.09 | 0.00 |
| *Trichosporon faecale* | 0.00 | 0.00 | 0.00 | 0.00 | 0.00 | 0.03 | 0.00 | 0.00 | 0.00 | 0.00 |
| *Ogataea cylindracea* | 0.02 | 0.07 | 0.00 | 0.00 | 1.48 | 0.00 | 0.00 | 0.00 | 0.00 | 0.00 |
| *Candida sake* | 0.00 | 0.00 | 0.00 | 0.00 | 0.00 | 0.00 | 3.56 | 1.45 | 0.00 | 2.19 |
| *Debaryomyces vindobonensis* | 0.00 | 0.00 | 0.00 | 0.00 | 0.00 | 0.00 | 0.01 | 0.00 | 0.00 | 0.01 |
| *Malassezia globosa* | 0.00 | 0.00 | 0.00 | 0.00 | 0.00 | 0.00 | 0.00 | 0.00 | 0.00 | 0.00 |
| *Cutaneotrichosporon guehoae* | 0.00 | 0.00 | 0.00 | 0.00 | 0.00 | 0.00 | 0.00 | 0.00 | 0.00 | 0.00 |
| *Candida parapsilosis* | 0.00 | 0.00 | 0.00 | 0.00 | 0.00 | 0.00 | 0.00 | 0.00 | 0.00 | 0.00 |
| *Meyerozyma guilliermondii* | 0.00 | 0.00 | 0.00 | 0.00 | 0.00 | 0.00 | 0.00 | 0.00 | 0.00 | 0.00 |
| *Trichosporon aquatile* | 0.00 | 0.00 | 0.00 | 0.00 | 0.00 | 0.00 | 0.00 | 0.00 | 0.00 | 0.00 |
| *Trichosporon lactis* | 0.00 | 0.00 | 0.00 | 0.00 | 0.00 | 0.03 | 0.00 | 0.00 | 0.00 | 0.00 |
| *Penicillium* | 0.00 | 0.03 | 0.09 | 0.02 | 0.00 | 0.00 | 0.00 | 0.00 | 0.00 | 0.01 |
| *Pichia cactophila* | 0.03 | 0.00 | 0.01 | 0.05 | 0.00 | 0.00 | 0.03 | 0.02 | 0.00 | 0.01 |
| *Rhodotorula mucilaginosa* | 0.00 | 0.00 | 0.00 | 0.00 | 0.00 | 0.00 | 0.00 | 0.00 | 0.00 | 0.00 |
| *Trichosporon ovoides* | 0.00 | 0.00 | 0.00 | 0.00 | 0.01 | 0.71 | 0.00 | 0.02 | 0.03 | 0.00 |
| *Candida davisiana* | 0.00 | 0.00 | 0.00 | 0.00 | 0.00 | 0.00 | 0.00 | 0.00 | 0.00 | 0.00 |
| *Actinomucor kuwaitiensis* | 0.00 | 0.00 | 0.00 | 0.00 | 0.00 | 0.00 | 0.00 | 0.00 | 0.00 | 0.00 |
| *Debaryomyces suglobosus* | 0.00 | 0.00 | 0.00 | 0.00 | 0.00 | 0.00 | 0.00 | 0.00 | 0.00 | 0.00 |
| *Fusarium* | 0.00 | 0.00 | 0.00 | 0.00 | 0.00 | 0.00 | 0.00 | 0.00 | 0.00 | 0.00 |
| *Skvortzovia furfurella* | 0.00 | 0.00 | 0.00 | 0.00 | 0.00 | 0.00 | 0.00 | 0.00 | 0.00 | 0.00 |

| Dairy Plant | E | E | E | E | E | E |
| --- | --- | --- | --- | --- | --- | --- |
| Code | F52 | F17 | F33 | F79 | F14 | F37 |
| Matrix | 5-d cheese | 5-d cheese | 5-d cheese | 15-d cheese | 15-d cheese | 15-d cheese |
| *Kluyveromyces marxianus* | 28.21 | 19.71 | 30.78 | 25.12 | 30.08 | 25.94 |
| *Galactomyces candidum* | 47.75 | 32.39 | 26.69 | 58.30 | 46.95 | 53.63 |
| *Galactomyces geotrichum* | 3.50 | 2.70 | 2.13 | 4.51 | 2.91 | 3.79 |
| *Geotrichum bryndzae* | 0.85 | 0.78 | 0.52 | 0.88 | 0.78 | 0.92 |
| *Galactomyces* | 0.55 | 0.40 | 0.23 | 0.49 | 0.36 | 0.61 |
| *Yarrowia lipolytica* | 1.88 | 10.70 | 5.40 | 2.99 | 3.80 | 5.07 |
| *Other* | 0.54 | 14.30 | 0.23 | 0.63 | 0.56 | 0.53 |
| *Trichosporon coremiiforme* | 9.75 | 7.91 | 32.78 | 1.22 | 9.77 | 8.15 |
| *Kurtzmaniella anglica* | 0.05 | 0.00 | 0.00 | 0.25 | 0.00 | 0.00 |
| *Debaryomyces hansenii* | 0.02 | 0.03 | 0.05 | 0.36 | 0.01 | 0.05 |
| *Saturnispora silvae* | 5.51 | 5.68 | 0.11 | 4.00 | 2.92 | 0.19 |
| *Penicillium roqueforti* | 0.00 | 0.02 | 0.01 | 0.00 | 0.01 | 0.01 |
| *Saccharomyces cerevisiae* | 0.12 | 0.84 | 0.00 | 0.09 | 0.03 | 0.11 |
| *Yarrowia* | 0.10 | 0.60 | 0.19 | 0.21 | 0.18 | 0.26 |
| *Trichosporon* | 0.24 | 0.29 | 0.43 | 0.03 | 0.15 | 0.22 |
| *Apiotrichum gracile* | 0.27 | 0.44 | 0.00 | 0.51 | 0.32 | 0.00 |
| *Cladosporium cladosporioides* | 0.01 | 0.02 | 0.01 | 0.01 | 0.01 | 0.01 |
| *Debaryomyces* | 0.00 | 0.00 | 0.00 | 0.03 | 0.00 | 0.00 |
| *Pichia fermentans* | 0.00 | 0.00 | 0.00 | 0.07 | 0.01 | 0.00 |
| *Scopulariopsis brevicaulis* | 0.00 | 0.00 | 0.00 | 0.00 | 0.00 | 0.00 |
| *Alternaria tenuissima* | 0.00 | 0.07 | 0.00 | 0.00 | 0.00 | 0.00 |
| *Cutaneotrichosporon curvatus* | 0.00 | 0.00 | 0.00 | 0.00 | 0.00 | 0.00 |
| *Kurtzmaniella santamariae* | 0.01 | 0.00 | 0.00 | 0.03 | 0.00 | 0.00 |
| *Torulaspora delbrueckii* | 0.02 | 0.00 | 0.00 | 0.02 | 0.05 | 0.00 |
| *Yarrowia deformans* | 0.01 | 0.40 | 0.06 | 0.05 | 0.48 | 0.10 |
| *Wickerhamiella pararugosa* | 0.02 | 0.01 | 0.01 | 0.00 | 0.01 | 0.01 |
| *Kazachstania unispora* | 0.12 | 0.00 | 0.00 | 0.04 | 0.01 | 0.00 |
| *Trichosporon faecale* | 0.17 | 0.10 | 0.25 | 0.01 | 0.12 | 0.08 |
| *Ogataea cylindracea* | 0.09 | 0.26 | 0.00 | 0.01 | 0.31 | 0.00 |
| *Candida sake* | 0.00 | 0.00 | 0.00 | 0.00 | 0.00 | 0.00 |
| *Debaryomyces vindobonensis* | 0.00 | 0.00 | 0.00 | 0.02 | 0.00 | 0.00 |
| *Malassezia globosa* | 0.00 | 0.02 | 0.00 | 0.00 | 0.00 | 0.00 |
| *Cutaneotrichosporon guehoae* | 0.00 | 0.00 | 0.00 | 0.00 | 0.00 | 0.00 |
| *Candida parapsilosis* | 0.00 | 0.01 | 0.00 | 0.00 | 0.00 | 0.00 |
| *Meyerozyma guilliermondii* | 0.00 | 0.00 | 0.00 | 0.00 | 0.00 | 0.00 |
| *Trichosporon aquatile* | 0.00 | 0.00 | 0.00 | 0.00 | 0.00 | 0.00 |
| *Trichosporon lactis* | 0.00 | 0.00 | 0.00 | 0.00 | 0.00 | 0.00 |
| *Penicillium* | 0.00 | 0.00 | 0.00 | 0.00 | 0.00 | 0.01 |
| *Pichia cactophila* | 0.05 | 0.05 | 0.01 | 0.00 | 0.00 | 0.00 |
| *Rhodotorula mucilaginosa* | 0.00 | 0.00 | 0.00 | 0.00 | 0.00 | 0.00 |
| *Trichosporon ovoides* | 0.02 | 0.11 | 0.06 | 0.00 | 0.05 | 0.19 |
| *Candida davisiana* | 0.00 | 0.00 | 0.00 | 0.00 | 0.00 | 0.00 |
| *Actinomucor kuwaitiensis* | 0.00 | 0.00 | 0.00 | 0.00 | 0.00 | 0.00 |
| *Debaryomyces suglobosus* | 0.00 | 0.00 | 0.00 | 0.00 | 0.00 | 0.00 |
| *Fusarium* | 0.00 | 0.01 | 0.00 | 0.00 | 0.00 | 0.00 |
| *Skvortzovia furfurella* | 0.00 | 0.00 | 0.00 | 0.00 | 0.00 | 0.00 |
